# Multi-level anomalous Hall resistance in a single Hall cross for the applications of neuromorphic device

**DOI:** 10.1038/s41598-020-58223-z

**Published:** 2020-01-28

**Authors:** Y.-U. Kim, J. Kwon, H.-K. Hwang, I. Purnama, C.-Y. You

**Affiliations:** Department of Emerging Materials Science, DGIST, Daegu, 42988 South Korea

**Keywords:** Ferromagnetism, Spintronics

## Abstract

We demonstrate the process of obtaining memristive multi-states Hall resistance (R_H_) change in a single Hall cross (SHC) structure. Otherwise, the working mechanism successfully mimics the behavior of biological neural systems. The motion of domain wall (DW) in the SHC was used to control the ascend (or descend) of the R_H_ amplitude. The primary synaptic functions such as long-term potentiation (LTP), long-term depression (LTD), and spike-time-dependent plasticity (STDP) could then be emulated by regulating R_H_. Applied programmable magnetic field pulses are in varying conditions such as intensity and duration to adjust R_H_. These results show that analog readings of DW movement can be closely resembled with the change of synaptic weight and have great potentials for bioinspired neuromorphic computing.

## Introduction

At present, the world is at the cusp of the 4th industrial revolution due to the recent surge in the use of neuromorphic computation such as machine learning^1–4^. However, conventional semiconductor-based electronic technology has been unable to provide the necessary hardware to support such high-level computation as CMOS technology, which has approached its fundamental technical limitation^5,6^. The amount of power consumption of cutting edge AlphaGo zero is about 1–2 kW, which is equivalent to the energy consumed by 10–20 humans^7^. However, performance reductions are also observed in the transition from AlphaGo Fan to AlphaGo zero. Furthermore, millions of simulated training games need to be played to sharpen skills like a human brain^8^. Oppositely, humans consumed about 100 W of power and only 20 W of it is used for biological brain activity^9,10^. Even if Moore’s Law continues to be realized, it will require about 30 years to have a comparable consumption^11,12^. Therefore, it is imperative that new technology is developed soon to satisfy the demand of future device requirements. The current density and power consumption of our device are 69.4 × 10^9^ A/m^2^ and 12 μW, respectively.

To solve such conundrum, domain wall (DW)-based spintronics technology has been proposed by the magnetics society as a possible candidate to replace semiconductor technology due to its non-volatility, high speed operation, and low power consumption^13–16^. For a DW-based device to be used in a neuromorphic circuit, it needs to possess several capabilities; first, it needs to show memristive behavior^17–19^; second, it needs to possess non-linear multi-state resistance^20,21^; third, its multi-state resistance must be time-sensitive to external stimulations^22–26^. In this study, we demonstrate that a single Hall cross (SHC) of typical perpendicular magnetic anisotropy (PMA) system (Co/Pt multilayer) is able to satisfy such necessary features and is therefore a good candidate for a neuromorphic device. In the SHC device, the neuromorphic features are accomplished via the motions of DW under the application of external magnetic field pulses with various duration and intensity. We show that a non-linear multi-level Hall resistance could be obtained and that it was sensitive to the sequence in which the external field pulses were applied. A set of complete field sweeps exemplify the memristive behavior of the SHC, which were verified by anomalous Hall effect (AHE) measurement and magneto-optical Kerr microscopy^27,28^.

## Result and Discussion

### Comparison of signals in the biological/artificial synaptic and DW device

**Figure 1:**
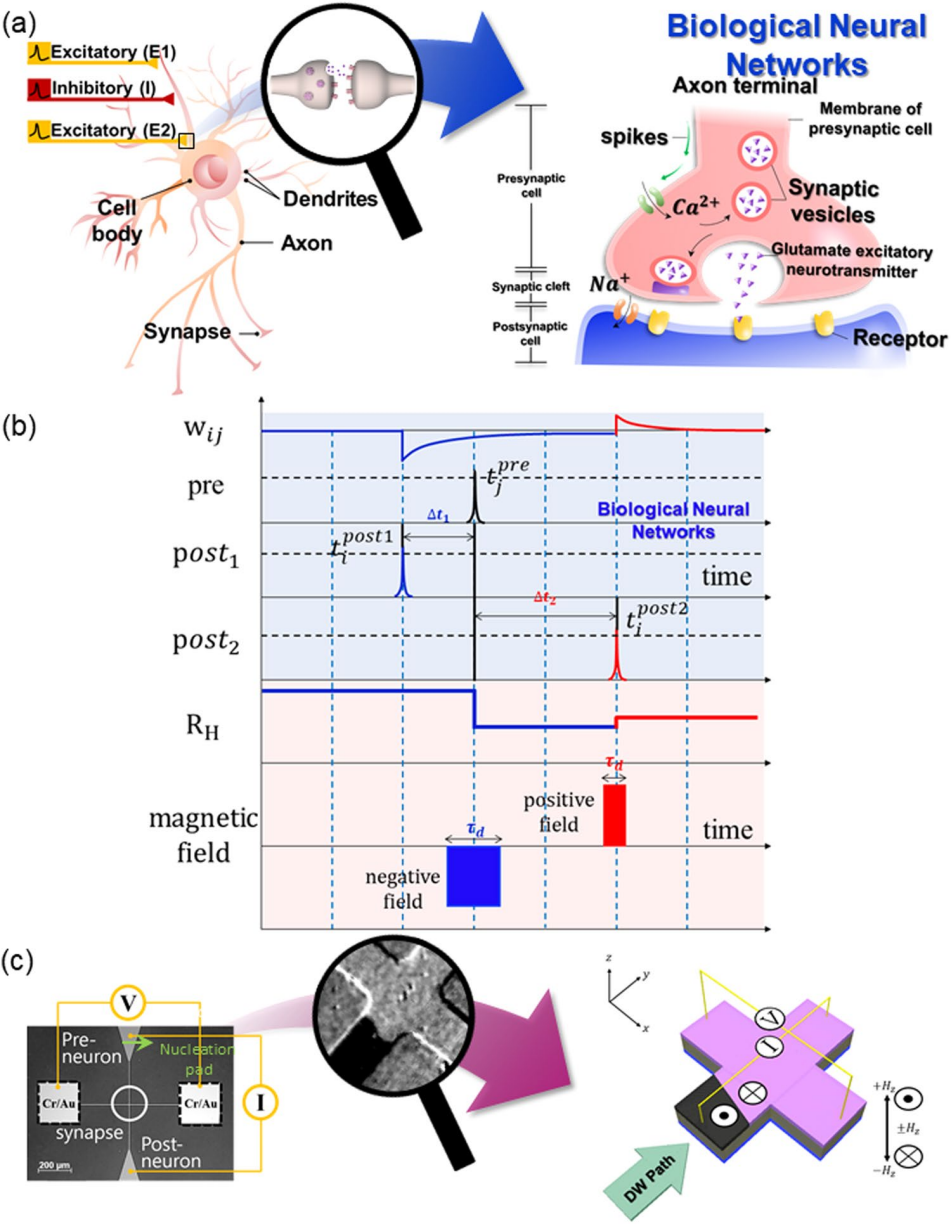
Utilizing a DW motion for imitation of artificial synapses. (**a**) The schematic representation of spikes transmission at the synapse, which is neuronal junction between pre- and postsynaptic neurons. (**b**) The schematic illustration of STDP which explained the dependence of w_ij_ on the spikes timing. R_H_ varies with adjusting magnetic field condition. (**c**) Kerr images of the SHC having wire width 10 μm. The illustration shows anomalous Hall resistance measurement set-up.

We first describe the likeness of SHC device inner workings to the mechanism of biological neural system. Figure 1(a) shows a schematic of a neuron, which is a basic component of a brain tissue. The neurons connected with each other through large numbers of synapses which are the junction between two neurons, where communications occur via neurotransmitter. The neurotransmitter travels across the synapse and tries to get the target neuron to increase or decrease its membrane potential. The synaptic weight between the neurons is then directly related to the membrane potential as well as the time interval between the signal that neuron receives and the signal that the neuron transmits^29,30^. This time-dependency of the synaptic weight is also known as spike-timing-dependent plasticity (STDP)^31,32^. Shown in the top part (blue shaded region) of Fig. 1(b) is the synapse connection strength (*w*_*ij*_) as a function of time. The *pre*, *post1*, and *post2*, correspond to the input signal of the neuron, and the two possible cases of output signals that the neuron exerts relative to the input signal. As can be inferred from the illustration, we can see that the synaptic strength will be decreased when the neuron exerts the output signal (*post1*) before the input signal (*pre*); on the other hand, the synaptic strength will be increased when the input signal (*pre*) comes before the output signal (*post2*). The decrease of the synaptic strength is appropriately termed as long-term depression (LTD), while the increase of the synaptic strength over a considerable amount of time is termed as long-term potentiation (LTP)^33,34^.

The bottom part (red shaded region) of Fig. 1(b) shows how the synaptic weight is represented as the Hall resistance (R_H_) reading in the SHC device. In particular, we show that the input and output signals are now represented by the external magnetic field pulses to z direction (*H*_*z*_); LTD now occurs due to the application of the negative *H*_*z*_, while LTP is excited through the application of positive *H*_*z*_. The time interval of the *pre* and *post* signals (*Δt*), which determines the extend of LTD and LTP in biological neuro system, is now represented by the duration and strength of the field pulses, with shorter *Δt* corresponding to stronger or longer field pulse.

Figure 1(c) shows the Kerr microscopy image of the SHC device. The layer structure of the SHC is Ta(4 nm)/Pt(3 nm)/[Co(0.6 nm)/Pt(6 nm)]_n=4_ with a capping layer of [Co(0.6 nm)/Pt (2 nm)] with two electrodes. Afterwards, the SHC was patterned to a wire width of 10 μm by the conventional photo-lithography process. Cr (5)/Au (100) electrode was patterned at a size of 50 μm^2^ for the electrical measurement. Two large triangular pads are also connected with two electrodes electrically. The DW was initially nucleated as a bubble-like domain at the triangular top pad. The bubble-like domains were then expanded by applying field pulses along the perpendicular direction of the sample plane which resulted in the DW being pushed into the SHC. Therefore, the top pad acts as the pre-neuron, where the information-carrying DW is nucleated. Inset shows the enlarged image of the SHC where a DW from the pre-neuron (top pad) travelled across the microwire and settled in the vicinity of the SHC. The dark area corresponds to the part where the magnetization of the microwire pointed along the +*z* direction (up domain). Afterwards, the DW motion in the Hall cross is detected by observing the change in the Hall resistance (ΔR_H_) and Kerr microscopy simultaneously. The more specific DW configuration can be investigated using the AHE method, which is simpler method in the fabrication process and allow direct measurement of the magnetization. As the DW moves forward within the SHC structure, multi-level resistances were observed at the SHC depends on the DW position or area of up domain. The relation between the multi-level R_H_ of the SHC and the DW position were then confirmed using the Kerr images^35–37^. Furthermore, the stimulus can be replaced by spin transfer torque and/or spin orbit torque, and the reading with TMR is also possible for the real devices improving cyclability, CMOS compatibility, and large ON/OFF ratio^38^.

**Figure 2:**
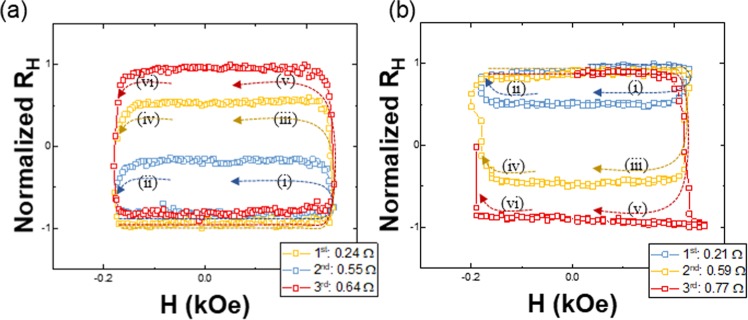
Memristive behavior with the magnetization of the SHC by sweeping field, following field sweeping sequence (i)-(vi). (**a**) The hysteresis shows multi-loops with increment of the R_H_ with initial saturation along –z-axis. (**b**) The decrease of the R_H_ can be achieved with initial saturation along the +z-axis.

For demonstration of memristive behavior of the SHC device, we show that the Hall resistance of the SHC, R_H_, changes according to the applied field history. The measured R_H_ values are normalized into the range [−1, 1] as shown in Fig. 2(a,b). The DW position was also observed by Kerr microscopy, simultaneously. Figure 2(a) shows the memristive behavior of the device when an up-down DW is present. The blue loop was obtained with a field sweep range from −180.4 Oe to 254.1 Oe. The yellow loop was obtained with a field sweep range from −174 Oe to 247.3 Oe. The red loop was obtained with a field sweep range from −178.6 Oe to 254.3 Oe. Figure 2(b) shows the memristive behavior of the device when a down-up DW is present. the sweeping fields for blue loop, yellow loop and red loop are in the range of 230 Oe~−180 Oe, 230 Oe~−200 Oe and 230 Oe~−210 Oe, respectively. By subjecting the SHC to three different field sweep history, we observe three different Hall resistance cycles which is the characteristic of memristor. It must be mentioned that the observed memristive behavior in this SHC device is different from our previous work^39^, where we reported the memristive behaviors in multiple Hall crosses.

### LTP and LTD based on the DW motion in the SHC

**Figure 3:**
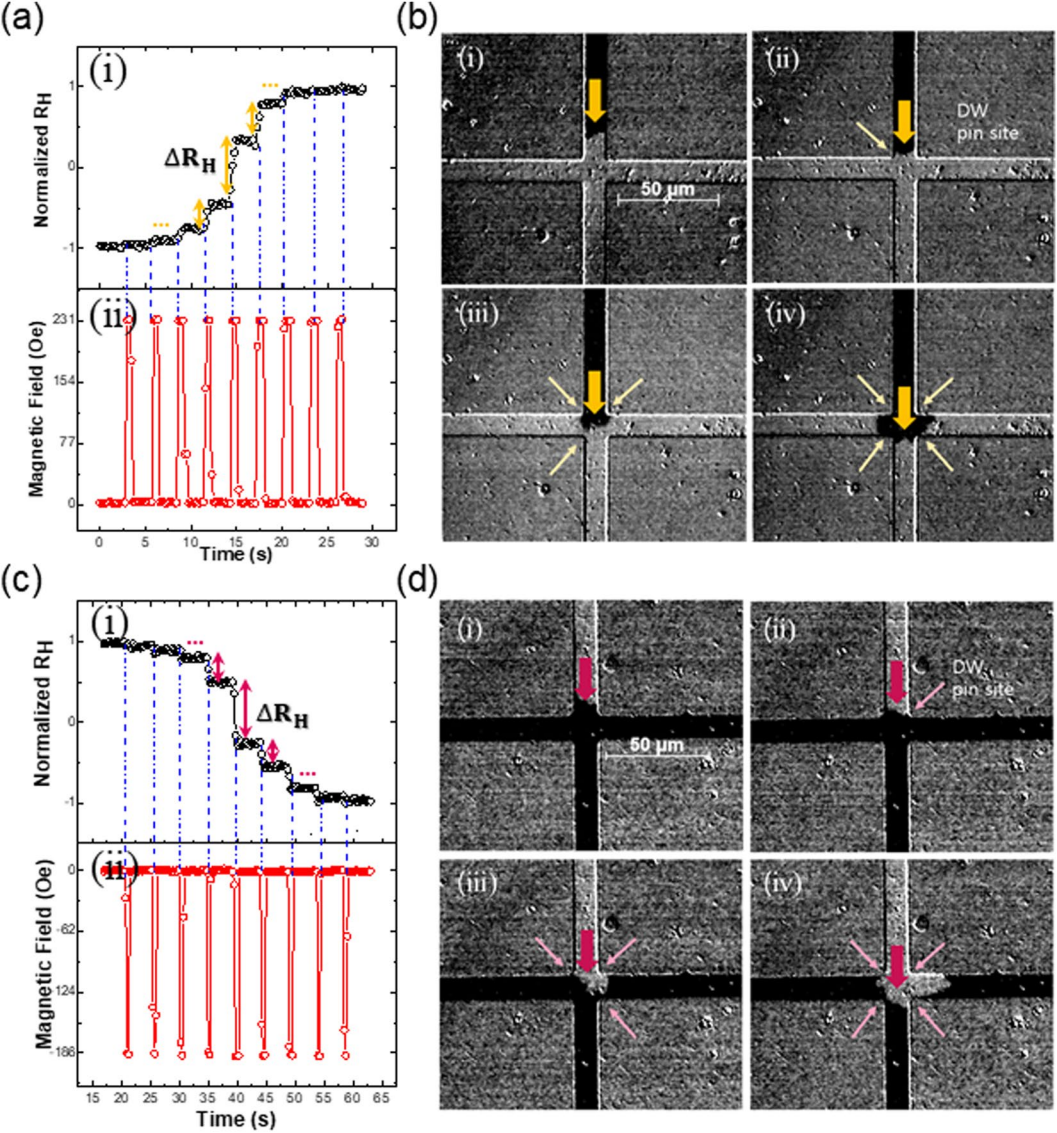
LTP and LTD processes relevant to the DW position in the SHC. (**a**) Multi-level during the R_H_ increases with UD-DW motion. (**b**) Kerr microscope shows the UD-DW motion related to LTP process in the increment of R_H_. (**c**) The DU-DW motion drives that the R_H_ decreases with multi-level and shows the LTD process. (**d**) The images of DU-DW passing via the SHC.

To demonstrate the synaptic capabilities of the device, first we show that the SHC is able to mimic the LTP and LTD behaviors. The analogue responses of R_H_ due to the DW motion in the SHC are shown in Fig. 3(a,c) in accordance with Kerr images shown in Fig. 3(b,d), respectively. Several levels of the R_H_ were obtained in the SHC during the DW propagation which emulates the LTP/ LTD of biological synaptic weights. The gradual increment/decrement of the R_H_ were obtained by the propagation of two different types of DWs; the increase in R_H_ is attributed to the movement of UD-DW, while the decrease in R_H_ is attributed to the movement of DU-DW, as shown in Fig. 3(c,d), respectively. To create the UD- DW/DU-DW, the structure was first saturated by $\mp$*H*_*z*_, which is greater than the coercivity (~320 Oe). Then, followed by an opposite field of ±*H*_*z*_ to nucleate and expand the Up/Down domains in the nucleation pad. We applied positive/negative pulses to position Up/Down domain at the entrance of the SHC. The successive field pulses with an intensity smaller than previous are applied to expand the Up/Down domains in the SHC, as shown in Fig. 3(b-i,d-i). Afterwards, the DWs are propagated along the SHC and the microwire via the application of driving field pulse at +231.8 Oe/−188.7 Oe with a duration time of 0.8 s/0.6 s. As can be seen from Fig. 3(b-ii,iii), the gradual expansion of the Up domain (dark) corresponds to the gradual increase in the normalized R_H_ value from −1 to +1. On the other hand, the gradual expansion of the Down domain (bright), as shown in Fig. 3(d-ii,iii) corresponds to the gradual decrease of the normalized R_H_ value from +1 to −1. After the application of 9 driving field pulses, the DWs left the SHC and no further change in the R_H_ was observed, as shown in Fig. 3(b-iv,d-iv).

### Paring duration dependence of STDP

**Figure 4:**
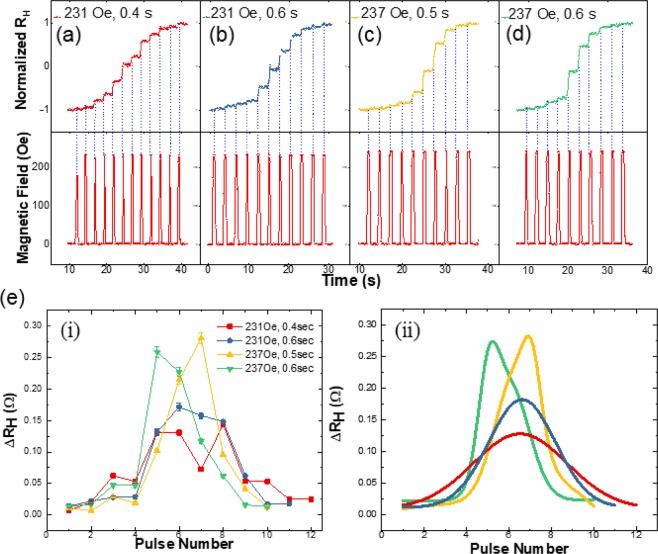
The change tendency of R_H_ versus the number of pulses. (**a**–**d**) The change of ΔR_H_ with a pulse train shows different number of levels in the R_H_ change. Each pulse conditions in a train are (**a**) intensity +231 Oe, duration 0.4 s, (**b**) +231 Oe, 0.6 s, (**c**) +237 Oe, 0.5 s, and (**d**) +237 Oe, 0.6 s. (**e**–**i**) Two parameters (intensity, duration) in a sequence of field pulses affect the ΔR_H_ as DW passes through the SHC. The quantitative analysis of ΔR_H_ peaks was performed by (ii) Gaussian fitting. The shape of ΔR_H_ is classified to the symmetrical (red, blue) and asymmetrical cases (yellow, green).

In addition to the multi-level feature of our device, we also show that the change in the potentiation and depression of the ΔR_H_ can be controlled using the amplitude and the duration of the field pulses. Figure 4(a–e) shows that by adjusting the amplitude and the duration time of the driving pulses, the change in the increment of R_H_, i.e. ΔR_H_ can be observed. Therefore, we investigate the distribution of ΔR_H_ during R_H_ change by applying identical field pulses in a train form (See the Supplementary Materials for additional details). For instance, in the UD-DW case, we applied pulse train with different amplitude and duration time as shown in Fig. 4(b–e). The result shows that ΔR_H_ increases gradually within ~10 levels with symmetrical ΔR_H_ as identical pulses are applied with amplitude +231 Oe and duration of 0.4 s in Fig. 4(b). When the amplitude of the pulses was increased to +237 Oe and the duration was increased to 0.5 s, an asymmetrical ΔR_H_ was observed as a function of the pulse sequence Fig. 4(d,e). Thus, the tendency of ΔR_H_ is classified into the two categories as shown in the Fig. 4(a–ii); 1) symmetrical case, which was obtained using low field amplitude and short pulse duration time; 2) asymmetrical case, which was obtained using high field and longer pulse duration time. In the symmetrical case, the gradual change in ΔR_H_ can be attributed to the more uniform DW propagation in the SHC. This also enables the relatively larger number of R_H_ states (~13 states) to be implemented at the SHC. The fitting result in Fig. 4(a–ii) indicates that the DW motion is reliable and uniform with the maximum peak height ΔR_H_ of 0.12 Ω, and 0.17 Ω while FWHM was 5.20, and 3.70 as shown in Fig. 4(a–i) red, blue curves.

On the other hand, the erratic change in ΔR_H_ in the asymmetrical case can be attributed to the disproportionate expansion of the DW within the SHC, Kerr image shows that the UD-DW is pinned and depinned from top-left and right edge corners in succession during the propagation process. In the initial steps (pulse 1–4), the ΔR_H_ was relatively small (<0.075), since the UD-DW is pinned to the two top corners of the SHC. The maximum value of ΔR_H_ is realized when the UD-DW is depinned from the top two corners and the up domain rapidly expands within the SHC (pulse 5–9). Afterward, the ΔR_H_ decreases sharply again, when the UD-DW is pinned by the bottom-right corner of the SHC (pulse 10–12). Asymmetrical forms with the ΔR_H_ changes are observed with the maximum peak height at ΔR_H_ 0.28 Ω and 0.27 Ω while the FWHM were 2.30 and 2.34 in Fig. 4(a–ii) yellow, green curves. The green line peaks have been observed through the depinning of UD-DW by top corner of the SHC when the 5^th^ field pulse is applied to the sample. The yellow peak could be obtained by the 7^th^ of pulse number. In the case of green, UD-DW moves along the wire faster, because the green line has the longer duration time than the yellow one.

### STDP learning rule of the SHC

**Figure 5:**
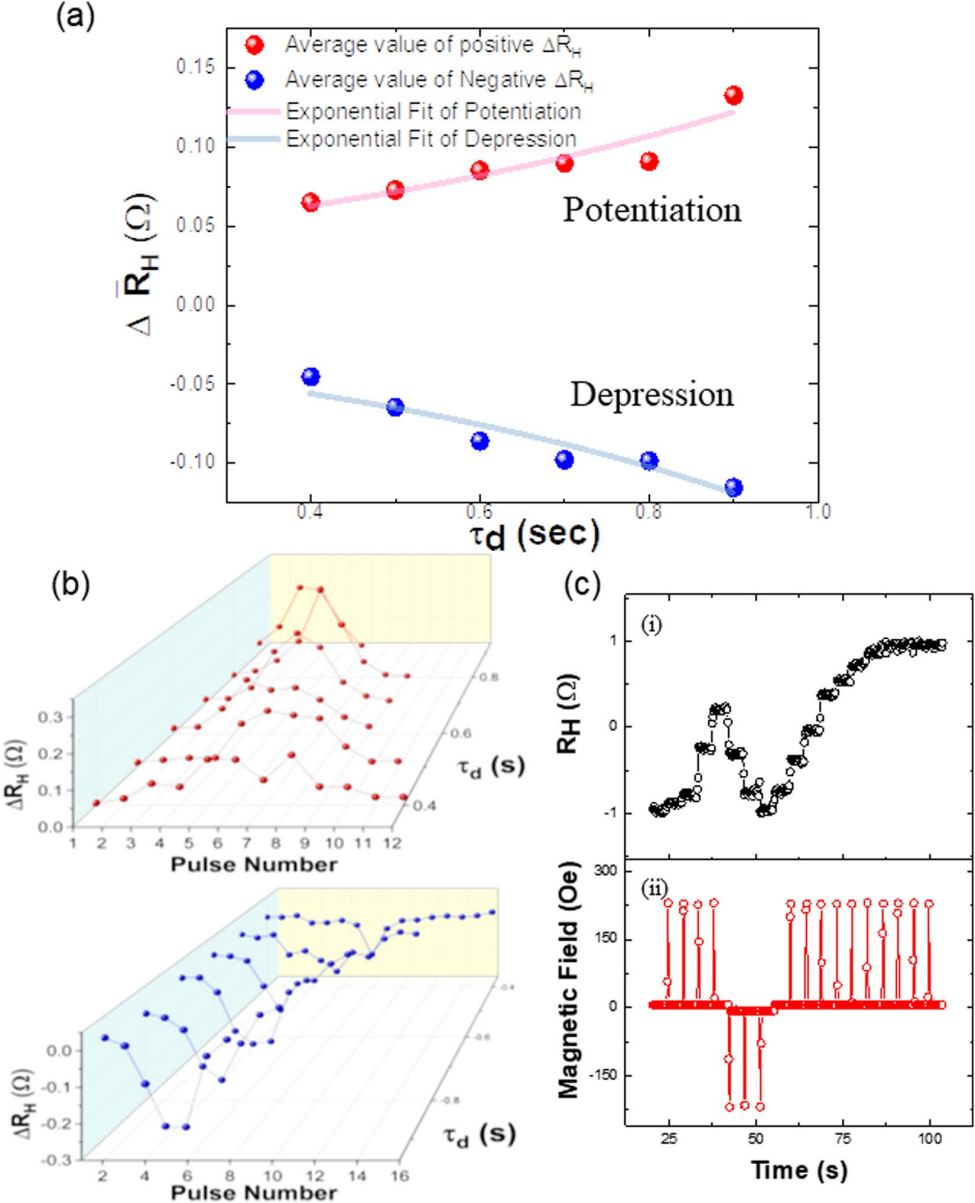
Change of ΔR_H_ related to pulse number with fixed intensity and duration 0.4 s in pulse trains. (**a**) The average ΔR_H_ ($\Delta {\bar{{\rm{R}}}}_{{\rm{H}}}$) as a function of duration. ${\Delta \bar{{\rm{R}}}}_{{\rm{H}}}$ is exponentially increased/decreased as shown in the exponential fit. (**b**) The 3D plot presents the change of ΔR_H_ as a function of duration and the number of pulses. Increasing duration and less pulse number (3–6^th^) has more chance to obtain large ΔR_H_. Result represents that the ΔR_H_ depends not only τ_d_ but also on the number of pulses. (**c**–**i**) The irregular shape of R_H_ achieves such as LTP. (ii) Bipolar pulse train applying with intensity +228, −217 Oe and duration 0.5 s for each pulse. Lastly, the direction of the saturation magnetization has been obtained R_H_ = +1.

As mentioned in the beginning, aside from the capabilities perform LTP and LTD, the SHC device is also capable to show the STDP capabilities which is one of the crucial aspects of a synaptic device. In the device, the pulse timing dependency is inversely represented by the pulse duration. For instance, in a standard biological synapse, shorter pulse interval between the pre- and post-synaptic spikes typically results in larger change in the synaptic weight; this feature is realized in the SHC device by applying pulses with stronger intensity or longer duration time. Figure 5(a) shows the relation between the average of the change in the Hall resistance (${\Delta \bar{{\rm{R}}}}_{{\rm{H}}}$) as a function of τ_d_. The average value is used here as in each case of τ_d_, the SHC undergoes various multi-state reading of R_H_. In the case of potentiation, a maximum ${\Delta \bar{{\rm{R}}}}_{{\rm{H}}}$ of 0.13 Ω was obtained when τ_d_ was set to 0.9 s. Similarly, for the case of depression, we obtained a maximum change of −0.11 Ω at τ_d_ = 0.9 s. In the nonlinear curve fitting, each ΔR_H_ slope is fitted to the exponential term ${\Delta R}_{{\rm{H}}}={{\rm{Ae}}}^{{{\rm{\tau }}}_{{\rm{d}}}/{\rm{b}}}$, where b values are 0.75 s and −0.67 s for potentiation and depression, respectively. The emulation of biological synapse function such as STDP would then be possible by combining a circuit which transforms spike timing information into field pulse duration information with the SHC device. The details of ΔR_H_ as a function of both pulse sequence and τ_d_ are shown in Fig. 5(b). As discussed previously, larger change in ΔR_H_ can be seen with larger τ_d_ for both potentiation and depression cases. By utilizing this information, we can then continuously perform the potentiation and depression to change the synaptic weight of the SHC device, as shown in top image of Fig. 5(c) as an example. The bottom image of Fig. 5(c) shows the corresponding field sequence, the applied field intensity was +228 Oe and −217 Oe, with the duration of each pulse fixed at 0.5 s. Here, the fixed pulse duration corresponds to a fixed time interval in STDP.

**Figure 6:**
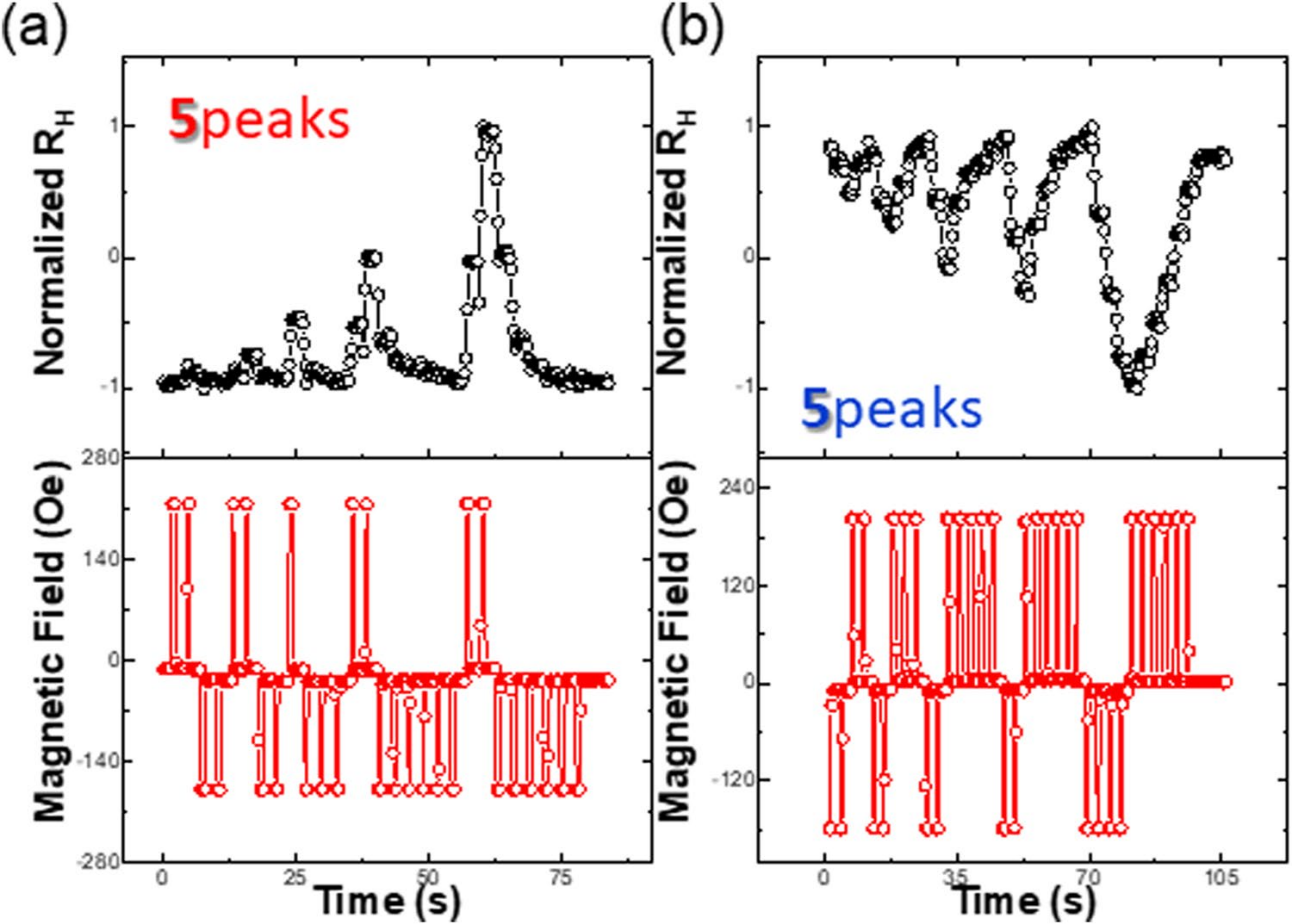
Potentiation and depression of R_H_ by controlling τ_d_ and pulse polarity in a pulse train sequence. (**a**) Potentiation of normalized R_H_ obtains through UD-DW motion by changing the τ_d_ of each pulse in the range from 0.4 s to 0.9 s and the altered field started with +236 Oe (altered field intensity −227 Oe) in a sequence. (**b**) The depression of R_H_ is obtained by controlling sequence of altered field launching with −177 Oe (altered field intensity +198 Oe).

The dependence of the multi-state Hall resistance of the SHC with respect to the various pulse duration time τ_d_ is shown in Fig. 6. As discussed before, longer τ_d_ corresponds to shorter time interval in the STDP scheme. Here, both Fig. 6(a,b) show that the positive and negative pulses trains with various τ_d_ can be used to adjust the potentiation and depression in the output signal. Alternating positive and negative pulses trains were employed to create five sharp and narrow R_H_ spikes with different peak values in Fig. 6 (a,b). The different values of R_H_ from the device depending on the applied pulse condition represent the capabilities of SHC to mimic the variable neural weight change in various spike timing different state.

## Conclusion

Contribution of this work may be summarized as follows. We have shown the formation of multi-state R_H_ obtained via the implementation of identical and non-identical field pulse with varying strength, polarity, and duration of pulses. It is worth noting that the input stimulus (pulses) amplitude and duration are below ~few hundred Oersted and milliseconds. Furthermore, the stimulus can be replaced by spin transfer torque and/or spin orbit torque, and the reading with TMR (tunneling magneto-resistance) is also possible for the real devices^40,41^. From device perspective, the use of DW and analogue R_H_ with modulated ΔR_H_ is potentially useful for cognitive and parallel computing in an artificial neuromorphic device. Overall, our device is simple, intuitive, and optimizable. We note that this work served to demonstrate the proof of concept experiments to realize such DW synapses for future ultralow-power intelligent neuromorphic system. Future work might be an implementation of on-chip cross-array, which would complete the building blocks towards integrated and biologically-inspired DW based neuromorphic device which is capable of adaptive STDP learning rule. In such device, the input to the postsynaptic neuron is determined by the multiplication of the output voltage of the presynaptic neuron and the synaptic weights in a crossbar array architecture. It has been shown that such architecture minimizes the power consumption for reading/writing and offers high connectivity and storage density that is comparable to previous CMOS-only hardware^42^. As for the concern regarding the value of the Hall resistance of our SHC device, the value is indeed much smaller compared to other technique such as the TMR reading of MTJ^43^, one possibility to circumvent this issue is by pairing the SHC device with a transistor, in similar spirit to how the signal is enhanced in a p-bit design^44^.

## Methods

### Film and patterned structure

The magnetic film of the SHC was deposited on the Si/SiO_2_ substrate using dc magnetron sputtering. The magnetic layers are comprised of Ta(4 nm)/Pt(3 nm)/[Co(0.6 nm)/Pt(6 nm)]_n=4_ with a capping layer of [Co(0.6 nm)/Pt (2 nm)]. In this structure, Ta layer acts as a buffer layer for the adhesion of the heavy metal to the Si/SiO_2_ substrate^45–47^. One of the advantages of our film structure (n ≥ 3) is that DWs can be easily nucleated and driven at low field range ~ 150–300 Oe due to structure defects, interface corners, and so on.

### Sample characterization

For a characterization of perpendicular magnetic anisotropy of multi-layered [Co/Pt]_n=4_, we used vibrating sample magnetometer (VSM) to measure magnetization hysteresis loop, which gave us information about total ~3 nm thick Co multi-layer in the stack at room temperature. After deposition, we patterned Hall bar into the sample by photolithography and ion milling.

### Experimental measurement

The Keithley 2400 Source meter and Keithley 2000 Multimeter has been used for measuring Hall resistance change in SHC. The constant current, 100 uA corresponding to the current density of 10^9^ A/m^2^ had been flows via current path in the SHC, and change of Hall voltage was monitored. A dc current flow has no effect on the DW motion driven by spin-transfer or spin-orbit torques^48,49^. The magnetic field pulses along out-of-plane are applied for DW in SHC to move by home-made solenoid magnet with NF Programmable AC/DC Power Source. All the measurements in this paper were conducted at room temperature.

## Supplementary information

Supplementary information.
