# Supplementary material for: Multi-level anomalous Hall resistance in a single Hall cross for the applications of neuromorphic device
Source: Sci Rep. 2020 Jan 28;10:1285. doi: 10.1038/s41598-020-58223-z (PMC6987114; doi:10.1038/s41598-020-58223-z)
Supplement: Supplementary file 1 — Supplementary information. [file 41598_2020_58223_MOESM1_ESM.docx]

**SUPPLEMENTARY MATERIALS**

**Multi-level anomalous Hall resistance in a single Hall cross for the applications of neuromorphic device**

Yoonui Kim, Jaesuk. Kwon, Hee-Kyeong Hwang, Indra Purnama, and Chun-Yeol You

*Department of Emerging Material Science, DGIST, Daegu 42988, South Korea*

**Table of contents**

**S1. Perpendicular magnetic anisotropy with multi-layered [Co/Pt]_n=4_**

**S2.** **The number of** $\mathbf{R}_{\mathbf{H}}$ **levels relevant to the intensity of pulses**

**S3.** **The number of** $\mathbf{R}_{\mathbf{H}}$ **levels relevant to the duration of pulses**

**S1. Perpendicular magnetic anisotropy with multi-layered [Co/Pt]**_n_

[Co/Pt]_n_ stacks are reported that anisotropy energy and coercivity are sensitive to the relevant Co layer thickness (t_Co_).^^[[1]](#endnote-1)^^ The interfacial effect is adapted to the uniaxial perpendicular anisotropy constant ($K_{u}$) in the multilayered Co/Pt film with an adjusted monolayer of the t_Co_. The configuration of domain wall (DW) with various $K_{u}$ varies in the xy plane.^^[[2]](#endnote-2)^^ In particular, [Co/Pt]_n=4_ stack shows that nucleation of multiple bubble domains occurs from inhomogeneities or defects.^[[3]](#endnote-3)^ Then, they are expanded along the microwire pattern in the formation of DW. The multiple bubble domains are nucleated conveniently from many defects and have the advantage for DW to be positioned in microwire.





**Figure S1.** (a) Hysteresis loop with normalized moment as a function of the out-of-plane (green) and in-plane (red) fields. The inset is a square hysteresis loop with perpendicular magnetic anisotropy due to the result in sweeping out-of-plane field. (b) Normalized anomalous Hall resistance (voltage) follows the square hysteresis with a constant current 100 μA as magnetic field is swept along out-of-plane.

For the [Co/Pt]_n=4_ stack, the in-plane and out-of-plane magnetization curves were measured by vibrating sample magnetometer (VSM), as shown in Fig. 1(a). The result shows that the effective anisotropy of the film is *K_eff_* = 651 kJ/m^3^, saturation magnetization *M_s_* = 1302.27 emu/cm^3^, and coercive field *H_c_* = ±321.75 Oe. The magnetization switching in the hysteresis loop indicates the Hall resistance ($R_{H}$) change of about Δ$R_{H}$ = ±1 $\Omega$ with the AHE measurement in Fig. 1(b). The change of $R_{H}$ is emerged from the propagation of a DW at the Hall cross area due to the application of external field pulses. For the AHE measurement. The application of constant current of 100 $\mu$A, in accordance with current density of ~ 1.3$\times$10^9^ A/m^2^. The current is small enough to be negligible for the Joule heating as well as not to be affected the DW motion.

**S2. The number of** $\mathbf{R}_{\mathbf{H}}$ **levels relevant to the intensity of pulses**

Multiple level of $R_{H}$ is obtained due to the external field pulses to drive a DW across the SHC. For the potentiation of $R_{H}$, intensity of pulse is modulated in variation with +234 Oe, +240 Oe, and +250 Oe, as shown in Fig. S2 (a-c) with a constant duration 0.3 s. From the results in Fig. S2 (a-c), number of levels in normalized $R_{H}$ has been counted with 12, 8, and 4. Afterward, the $R_{H}$ reaches to saturation with the maximum values of +1.

For the depression case in Fig. S2 (d-e), the field has applied with intensity -202.6 Oe, -214.7 Oe, and -219.5 Oe, which corresponds to the number of levels in 19, 8, and 4. The saturation occurs that normalized $R_{H}$ decreases to -1 and remains the saturation value with applied extra field pulses. The large number of levels (~ 19) enables to drive non-linear $R_{H}$ change. The results are obtained in the SHC, and large number of levels and nonlinearity (analogue signals) is favorable for the application of neural network computation.





**Figure S2.** Normalized $R_{H}$ as a function of time with following conditions: pulse duration of 0.3 s and interval time of 4 s between two pulses. Potentiated $R_{H}$ having (a) 13 levels with pulse intensity +234 Oe, (b) 8 levels with +240 Oe, and (c) 4 levels with +250 Oe. Depressed $R_{H}$ having (d) 19 levels during executed negative pulses with intensity -203 Oe, (e) 8 levels with -215 Oe, and (f) 4 levels with -220 Oe.

**S3. The number of** $\mathbf{R}_{\mathbf{H}}$ **levels relevant to the duration of pulses**

$R_{H}$ is adjustable due to the various duration time of field pulses. Fig. S3 (a-f) shows the variation of $R_{H}$ as the duration is applied from 0.4 s to 0.9 s.





**Figure S3.** Varying $R_{H}$ with increasing duration. Positive and negative field intensities are fixed with +228 Oe / -173 Oe, respectively. As well, interval between pulses is set to be 2 s. Potentiated $R_{H}$ shows (a) 13 levels with pulse duration 0.4 s, (b) 10 levels with duration 0.6 s, and (c) 7 levels with duration 0.9 s. Depressed $R_{H}$ has (d) 17 levels during negative pulses with duration 0.4 s, (e) 13 levels with duration 0.5 s, and (f) 8 levels with duration 0.9 s.

As mentioned in the manuscript, the pulse duration is linked to the time interval between the pre- and post- synaptic spikes in biological brain. Longer pulse duration in the SHC device represents a shorter time interval between the pre- and post- spikes, while short pulse duration represents longer time interval in fixed total time measurement. The result indicates that a SHC device has a possibility to use as a memristor for neuromorphic applications with spike-timing-dependent-plasticity (STDP) function.^[[4]](#endnote-4),^ ^[[5]](#endnote-5)^

**References**

1. Lin, C.-J. *et al.* Magnetic and structural properties of Co/Pt multilayers. *J. Magn. Magn. Mater.* **93***,* 194 (1991). [↑](#endnote-ref-1)
2. Chureemartm P., Evans, R. F., D'Amico, I., and Chantrell R. W. Influence of uniaxial anisotropy on domain wall motion driven by spin torque. *Phys. Rev. B.* **92**, 054434 (2015). [↑](#endnote-ref-2)
3. Vogel, J., Moritz, J., Fruchart, O. Nucleation of magnetisation reversal, from nanoparticles to bulk materials. *CR PHYS.* **7**, 977 (2006). [↑](#endnote-ref-3)
4. Brette, R. and Gerstner, W. Adaptive Exponential Integrate-and-Fire Model as an Effective Description of Neuronal Activity. *J. Physiol.* **94**, 3637 (2005). [↑](#endnote-ref-4)
5. Snider, G. Spike-timing-dependent learning in memristive nanodevices. *2008 IEEE/ACM International Symposium on Nanoscale Architectures*. Washington, DC, USA, pp. 85-92 (2008). [↑](#endnote-ref-5)
